# Amine Gas‐Induced Reversible Optical Bleaching of Bismuth‐Based Lead‐Free Perovskite Thin Films

**DOI:** 10.1002/advs.202306391

**Published:** 2023-12-03

**Authors:** Fuxiang Ji, Bin Zhang, Weimin M Chen, Irina A Buyanova, Feng Wang, Gerrit Boschloo

**Affiliations:** ^1^ Department of Chemistry‐Ångström Laboratory Physical Chemistry Uppsala University Uppsala SE‐751 20 Sweden; ^2^ Department of Physics Chemistry and Biology (IFM) Linköping University Linköping SE‐58 183 Sweden

**Keywords:** Cs_2_AgBiBr_6_, Cs_3_Bi_2_I_9_, lead‐free perovskites, methylamine gas, optical bleaching, smart windows

## Abstract

Reversible optical property changes in lead‐free perovskites have recently received great interest due to their potential applications in smart windows, sensors, data encryption, and various on‐demand devices. However, it is challenging to achieve remarkable color changes in their thin films. Here, methylamine gas (CH_3_NH_2_, MA^0^) induced switchable optical bleaching of bismuth (Bi)‐based perovskite films is demonstrated for the first time. By exposure to an MA^0^ atmosphere, the color of Cs_2_AgBiBr_6_ (CABB) films changes from yellow to transparent, and the color of Cs_3_Bi_2_I_9_ (CBI) films changes from dark red to transparent. More interestingly, the underlying reason is found to be the interactions between MA^0^ and Bi^3+^ with the formation of an amorphous liquefied transparent intermediate phase, which is different from that of lead‐based perovskite systems. Moreover, the generality of this approach is demonstrated with other amine gases, including ethylamine (C_2_H_5_NH_2_, EA^0^) and butylamine (CH_3_(CH_2_)_3_NH_2_, BA^0^), and another compound, Cs_3_Sb_2_I_9_, by observing a similar reversible optical bleaching phenomenon. The potential for the application of CABB and CBI films in switchable smart windows is investigated. This study provides valuable insights into the interactions between amine gases and lead‐free perovskites, opening up new possibilities for high‐efficiency optoelectronic and stimuli‐responsive applications of these emerging Bi‐based materials.

## Graphical abstract

Methylamine gas (MA^0^) induced switchable optical bleaching of lead‐free bismuth‐based halide perovskites through phase transformation is demonstrated for the first time. The color of Cs_2_AgBiBr_6_ and Cs_3_Bi_2_I_9_ films reversibly changes from yellow and red to transparent. MA^0^ molecules interact with Bi^3+^, transforming the colored perovskite phase into a transparent amorphous non‐perovskite phase. Subsequent recrystallization gives improved grain orientation and morphology.

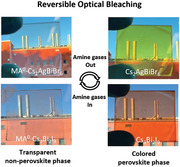

## 1 Introduction

Lead halide perovskites have brought a revolution in the optoelectronic field over the past decade owing to their unique optical and electronic properties. However, they suffer from intrinsic metastability issue, which is mainly related to their soft crystal nature and low formation energies.^[^ ^1^, ^2^, ^3^, ^4^ ^]^ Interestingly, these unique characteristics enable them to undergo reversible phase (chemical and structural) transformation by exposure to certain external stimuli, including temperature, pressure, chemical environment, etc., which could reversibly switch their optical and electrical properties.^[^ ^5^, ^6^ ^]^ This extends lead halide perovskites applications beyond photovoltaics, such as smart windows,^[^ ^7^ ^]^ sensors,^[^ ^8^ ^]^ memory devices,^[^ ^9^ ^]^ and data encryption.^[^ ^10^ ^]^ For example, the brown (CH_3_NH_3_)_4_PbI_6_ perovskite films can be changed to transparent after absorbing water molecules with the formation of transparent (CH_3_NH_3_)_4_PbI_6_·2H_2_O film which would be converted to (CH_3_NH_3_)_4_PbI_6_ again after the dehydration process by heating.^[^ ^11^ ^]^ Different from the combination with H_2_O, the color change of all‐inorganic CsPbI_3−x_Br_x_ perovskite is due to the thermally‐driven, moisture‐mediated reversible transitions between a colored perovskite phase and a transparent non‐perovskite phase.^[^ ^7^ ^]^ Thanks to their reversible optical changes, smart photovoltaic windows based on these lead‐based perovskite films were further demonstrated. Another interesting stimuli employed for lead‐based perovskites are various chemical environments induced by different gas vapors, such as ammonia (NH_3_),^[^ ^12^, ^13^ ^]^ methylamine (CH_3_NH_2_, MA^0^),^[^ ^14^, ^15^, ^16^ ^]^ pyridine,^[^ ^17^ ^]^ methanol,^[^ ^18^ ^]^ etc. Through the absorption and release of gas molecules, a reversible phase transition of perovskites is induced, which could significantly modulate their visible light absorption.

Motivated by these interesting observations in lead‐based perovskites, it is valuable to explore switchable optical properties in non‐toxic and stable lead‐free perovskites considering practical applications. Gao and coworkers first reported the temperature‐induced reversible optical properties changes in double perovskite Cs_2_AgBiBr_6_ (CABB), where its crystal color changes from red to black as the temperature increases from room temperature to 250 °C.^[^ ^19^ ^]^ Later, a similar behavior was observed in A_3_B^3+^_2_X_9_‐type perovskites (we also refer to these A_3_B^3+^_2_X_9_‐type perovskite derivatives as perovskites in this study for simplicity). The crystal colors of Cs_3_Sb_2_I_9_ and Cs_3_Sb_2_Br_9_ change from red to black within a temperature range of 30–140 °C and from yellow to red over a range of 30–210 °C, respectively.^[^ ^20^, ^21^ ^]^ The underlying thermochromism mechanisms for these lead‐free materials are the combined effect of electron‐phonon coupling, spin‐orbit coupling, and lattice expansion.^[^ ^19^, ^20^, ^21^, ^22^ ^]^ Following this line, we achieved the most significant color change from light yellow to black in lead‐free halide perovskites to date by using Cs_2_NaFeCl_6_ with strong electron‐phonon coupling.^[^ ^23^, ^24^ ^]^ The temperature sensitivity of the bandgap in Cs_2_NaFeCl_6_ is up to 2.52 meVK^−1^, and the bandgap change from −266 to 150 °C is 0.51 eV. Unfortunately, the remarkable color changes exhibited in these crystals become much weaker and even negligible in their thin films, primarily due to their low absorption coefficients. Together with the high temperature (140–250 °C) required for color variation, these key issues create significant challenges for further potential applications of these materials, such as smart windows.

Herein, we find MA^0^ gas‐induced reversible and dramatic color changes from transparent to yellow and red in CABB and CBI thin films, respectively. The experimental characterizations indicate that the MA^0^ gas molecules can destroy the original crystal structure of the perovskite thin films with the formation of a transparent intermediate non‐perovskite phase. Interestingly, the absorbed MA^0^ gas can be completely removed from the film at relatively low temperatures (50–75 °C), which results in the recrystallization of the intermediate phase back to the original perovskite phase with improved grain orientation and film morphology. To the best of our knowledge, this is the first time to report phase transition‐induced color variation in lead‐free perovskite thin films. This reversible optical bleaching behavior is further explored as switchable smart windows, revealing its potential applicability in various on‐demand applications.

## 2 Results and Discussion

**Figure 1:**
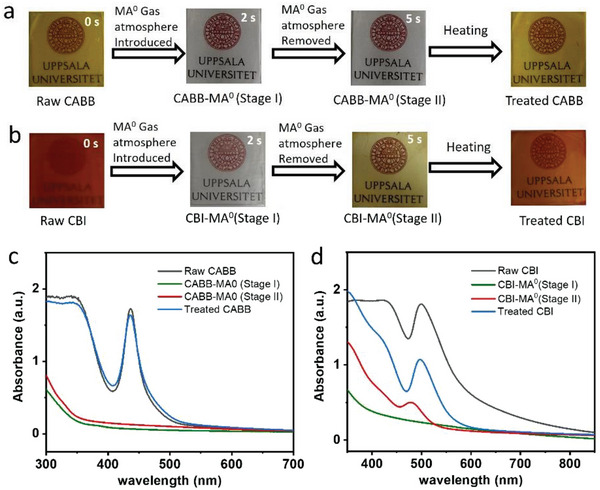
Photographs of optical bleaching in CABB a) and CBI films b) during MA^0^ gas treatment. UV–vis absorption spectra of CABB c) and CBI d) film at different stages during MA^0^ gas treatment.

Both CABB and CBI thin films on a glass substrate were fabricated through a one‐step spin coating method in an ambient environment with nitrogen gas flow. Optical photographs in **Figure** **1** demonstrate the MA^0^ gas‐induced optical bleaching in Bi‐based perovskite films. It can be seen that the yellow color of the CABB film changed significantly to transparent after being exposed to MA^0^ gas atmosphere for a few seconds, which can be termed as Stage I. After removing the film from the MA^0^ gas atmosphere, the CABB film retains its transparent appearance, we refer to this film stage as Stage II. Under further annealing, the film returns to its original yellow color, indicating a completely reversible process (Figure 1a). Similarly, the red CBI film quickly becomes transparent (Stage I) under MA^0^ gas atmosphere. The major difference is that the CBI film first turns light yellow (Stage II) after being removed from the MA^0^ gas environment, implying that the release of MA^0^ molecules is much easier in CBI than in CABB. Further annealing accelerates the gas desorption process with the color change of the CBI film back to red but slightly lighter than the original film (Figure 1b). Notably, the color recovery process of CABB and CBI films can also occur automatically at room temperature, taking ≈70 h and 1 h, respectively (Figure S1, Supporting Information).

UV–vis measurements are performed to quantify the film color changes for the optical bleaching processes. As shown in Figure 1c, raw CABB film shows typical absorption of CABB perovskite with a shape absorption edge at ≈470 nm and a characteristic peak at ≈437 nm.^[^ ^25^ ^]^ After the CABB film exposure to MA^0^ gas atmosphere (Stage I), there is almost no light absorption in visible range, consistent with the transparent color. The Stage II CABB film showed a very similar absorption spectrum to Stage I, with slightly increased absorbance. After fully removing the MA° from the film, the treated CABB film recovers the same absorption feature as the raw film, confirming the reversible process. Figure 1d shows the absorption changes of CBI film during the MA^0^ treatment process. The raw CBI film shows a sharp absorption edge at ≈600 nm and a strong tail absorption.^[^ ^26^ ^]^ These features totally disappear after the optical bleaching process by immersing the raw film in an MA^0^ atmosphere (Stage I). After removing the film from the MA^0^ atmosphere (Stage II), a weak characteristic absorption peak at ≈480 nm appeared quickly due to the partially desorbed MA^0^ gas molecules, consistent with the yellow film color. All original absorption features appear again after completely removing the MA^0^ gas in the film by annealing. Notably, the absorbance of the characteristic peak at 500 nm decreased by half after the MA^0^ gas treatment (Figure 1d), which is mainly related to the thickness and morphological changes in films that will be discussed later.

**Figure 2:**
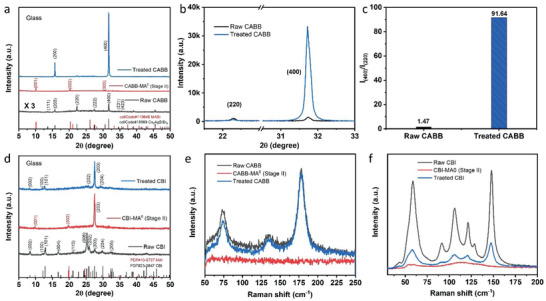
a) XRD patterns of CABB film at different stages during MA^0^ treatment. The diffraction intensity of raw CABB film is multiplied three times for better comparison. b) The enlarged view of the (220) and (400) diffraction peaks in the XRD patterns of raw and MA^0^‐treated CABB films. c) The diffraction intensity ratio between the (400) and (220) peaks in raw and MA^0^‐treated CABB films. d) XRD patterns of CBI film at different stages during MA^0^ treatment. Raman spectra of CABB e) and CBI f) film at different stages during MA^0^ treatment.

To understand the mechanisms of the optical bleaching, we perform X‐ray diffraction (XRD) measurements and Raman spectroscopy to investigate structural changes during the MA^0^ treatment. As shown in **Figure** **2**a, the raw CABB exhibits a typical CABB double perovskite phase with randomly oriented grains.^[^ ^27^ ^]^ After the MA^0^ gas treatment (Stage II CABB film), all perovskite diffraction peaks disappear, indicating that the initial double perovskite phase is entirely transformed into an amorphous non‐perovskite phase. This causes the film to become transparent. Notably, we observe three new diffraction peaks belonging to MABr appearing in this Stage II film. This is possibly due to the presence of a tiny amount of H_2_O during the treatment process and XRD measurement process, which could react with MA^0^ to produce MA^+^ cations in the film.^[^ ^28^ ^]^ These peaks completely disappeared after annealing the film, and the CABB double perovskite phase reformed. Impressively, the treated CABB film shows a significantly enhanced crystallinity with the dominant (400) diffraction peak 26 times stronger than in the raw CABB film (Figure 2a,b). Moreover, the diffraction intensity ratio between the (400) and (220) diffraction peaks (I_(400)/_I_(220)_) is dramatically increased from 1.47 to 91.64, indicating the grain orientation of CABB film becomes almost uniaxially oriented with diffractions of the {200} peak series (Figure 2c). We also noticed that this behavior is highly substrate‐dependent; that is, the significantly enhanced XRD intensities and grain orientation are suppressed when using other substrates such as ITO, FTO, and TiO_2_ (Figure S2, Supporting Information). This could be related to the crystallinity of the substrate since only glass is the amorphous substrate.

A similar optical bleaching phenomenon is also observed in the CBI film. As shown in Figure 2d, the raw CBI film shows a typical XRD pattern same as the previous reports.^[^ ^26^ ^]^ Under MA^0^ atmosphere, the transparent intermediate state (Stage I) is formed. The film starts recrystallization (Stage II) after removal from the MA^0^ atmosphere due to the quickly released absorbed MA^0^ molecules. In this stage, three diffraction peaks appear in the film, two of which belong to MAI and one from CBI (Figure 2d), suggesting that the CBI is much easier to recrystallize than CABB. Upon heating, MA^0^ gas release is expedited, resulting in the disappearance of two MAI diffraction peaks and the emergence of more CBI diffraction peaks, as shown in Figure 2d. Meanwhile, the preferred film orientation of the CBI film changed from (006) to (203) after MA^0^ treatment. Different from the CABB film, the intensity of diffraction peaks in the MA^0^‐treated CBI film did not exhibit an enhancement. This is possibly because it is more challenging to recrystallize CBI with a low structural dimension (0D) than CABB (3D) in one preferred orientation.

The structure change behavior during MA^0^ treatment is further confirmed by Raman spectroscopy. For the raw CABB film, three clear Raman peaks at 73, 136, and 178 cm^−1^ are observed at room temperature (Figure 2e), corresponding to internal modes of the octahedral.^[^ ^29^, ^30^ ^]^ After exposure to MA^0^ gas (Stage II), all Raman peaks disappear, confirming the collapse of the structure of CABB. As expected, these peaks are fully recovered after removing MA^0^ from the film due to the reformation of the perovskite structure, indicating the fully reversible process. As for CBI film, the Raman spectrum has six major peaks at 58.8, 91.6, 106.1, 121.2, 128.4, and 147.9 cm^−1^, respectively (Figure 2f). Amongst, the three peaks with the high energy (121.2, 128.4, and 147.9 cm^−1^) come from terminal Bi─I symmetric and asymmetric stretch. The peaks at 106.1 and 91.6 cm^−1^ belong to the symmetric and asymmetric stretch of bridge Bi─I, respectively. The lower energy peak at 58.8 cm^−1^ corresponds to the Bi─I bending mode.^[^ ^31^ ^]^ After treatment with MA^0^ gas, only weak and broad Raman peaks emerged in the Stage II CBI film (Figure 2f), which is consistent with the observed immediate recrystallization in Figure 2d. By completely releasing the MA^0^ gas from the film, the characteristic Raman peaks of CBI are fully recovered with decreased intensity. This is also correlated with the reduced absorbance of the treated CBI film (Figure 1d).

**Figure 3:**
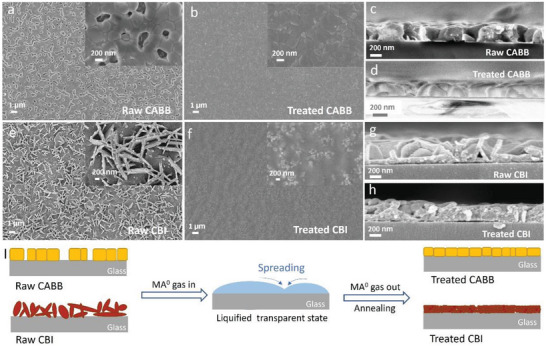
Surface SEM images of raw CABB a) and MA^0^‐treated CABB b) film. A higher magnification is presented in the inset for each sample. Cross‐sectional SEM images of raw CABB c) and MA^0^‐treated CABB d) film. Surface SEM images of raw CBI e) and MA^0^‐treated CBI f) film. Cross‐sectional SEM images of raw CBI g) and MA^0^‐treated CBI h) film. e) Schematic illustration of MA^0^ gas treatment process for Bi‐Based perovskite thin films.

We characterize the morphology evolutions of these Bi‐based perovskite thin films during MA^0^ treatment through scanning electron microscopy (SEM) imaging. As shown in **Figure** **3**a, the raw CABB film shows inhomogeneous morphology with many pinholes. Impressively, a dense and smooth CABB film is formed after MA^0^ gas treatment, indicating that the collapsed crystal structure leads to a liquefied non‐perovskite phase, which can self‐spread to cover the pinholes in the raw film. The morphology changes are also confirmed by the cross‐sectional SEM images in Figure 3c,d. For the CBI film, its morphology also significantly improved after MA^0^ gas treatment. As shown in Figure 3e,g, the raw CBI film exhibits a typical rough surface with thin flakes standing perpendicular to the glass substrate, consistent with previous reports.^[^ ^26^ ^]^ After MA^0^ gas treatment, these flake structures collapse and finally form a dense film with small grains (Figure 3f,h), which is attributed to the formation of a liquefied intermediate state. This remarkable morphology change, along with a reduced film thickness in the MA^0^‐treated CBI film could cause an intensity reduction in both absorbance (Figure 1d) and Raman spectrum (Figure 2f). For clarity, the MA^0^ treatment process of Bi‐based perovskite films is schematically illustrated in Figure 3e, where MA^0^ gas molecules collapse the initial solid film to a liquefied intermediate state that can self‐cover the pinholes and recrystallize back to a solid state with the original perovskite structure after full removal of the absorbed MA^0^ gas. This process significantly changes the initial rough morphology into a more uniform one, which is highly beneficial for various optoelectronic applications.

**Figure 4:**
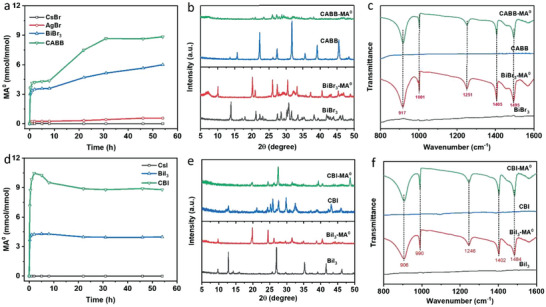
a) MA^0^ gas absorption behavior of different halide salts and perovskite powder in CABB, including CsBr, AgBr, BiBr_3_, and CABB powder samples. XRD patterns b) and FTIR spectra c) of BiBr_3_ and CABB powder before and after absorbing MA^0^ gas molecules. d) MA^0^ gas absorption behavior of different halide salts and perovskite powder in CBI, including CsI, BiI_3_, and CBI powder samples. XRD patterns e) and FTIR spectra f) of BiI_3_ and CBI powder before and after absorbing MA^0^ gas molecules.

To gain further insight into the mechanism of the optical bleaching processes, we investigate the interaction between Bi‐based perovskite and gas molecules by exposing different halide salts and perovskite powders, including CsBr, BiBr_3_, AgBr, CABB, CsI, BiI_3_, and CBI, with the MA^0^ gas. By balancing the mass differences of powders with different gas exposure times, we can calculate the amount of absorbed MA^0^ molecules (x mol MA^0^ mol^−1^ halide salts or perovskite material) as a function of time. In **Figure** **4**a and Figure S3 (Supporting Information), it is observed that the CABB powder rapidly absorbs a large amount of MA^0^ gas (≈4.14 mmol mmol^−1^) within the first 1 h, and finally reaches the maximum saturation amount of 8.8 mmol mmol^−1^. Notably, BiBr_3_ shows a saturation MA^0^ amount of 6.0 mmol mmol^−1^, which is close to that of CABB powder. Besides, AgBr could absorb trace amounts of MA^0^ molecules (0.57 mmol mmol^−1^), while CsBr powder has a negligible MA^0^ absorption ability. These observations indicate that the MA^0^ absorption behavior in CABB mainly arises from the interactions between MA^0^ and Bi^3+^. As mentioned before, thin films can form a liquefied amorphous intermediate phase after exposure to the MA^0^ gas due to their small thickness (≈300 nm) and large contact area with MA^0^ molecules. The solid powders can only transform into a paste rather than a liquid under similar conditions (room temperature and normal pressure), with a color change from brown and yellow to white for CABB and BiBr_3_, respectively (Figure S4, Supporting Information).

By further checking their structural changes after absorbing MA^0^ gas molecules through XRD analysis, we find the XRD patterns of CsBr and AgBr powder samples do not exhibit any change, which indicates that there is no interaction between these materials and MA^0^ molecules (Figure S5, Supporting Information). In contrast, BiBr_3_ and CABB powder samples show significant XRD changes with the original diffraction peaks disappearing (Figure 4b), verifying the interactions between the MA^0^ and Bi^3+^. In addition, similar final XRD patterns appear in CABB and BiBr_3_ powder samples after absorbing MA^0^ molecules, which mainly belong to the byproduct MABr (Figure 4b; Figure S6, Supporting Information), corresponding well with the observation in MA^0^‐treated films (Figure 2a). The interactions of MA^0^ and Bi^3+^ are further confirmed by the Fourier transform infrared spectroscopy (FTIR). As shown in Figure S7 (Supporting Information), no FTIR features are detected in CsBr and AgBr powders before and after MA^0^ treatment. While some new features at 917, 1001, 1251, 1405, and 1495 cm^−1^ are observed in MA^0^‐treated CABB and BiBr_3_ powders, which are consistent with the byproduct MABr (Figure 4c).

We performed the same experiment to study the absorption behavior of CBI and its precursors, including CsI, BiI_3_, and CBI powder samples. Similar to CABB, only BiI_3_ has a good MA^0^ absorption ability with a saturation amount of 4.30 mmol mmol^−1^, while CsI does not absorb MA^0^ molecules. CBI shows a high saturation MA^0^ amount of 10.47 mmol mmol^−1^ (Figure 4d). It is worth noting that the main absorption process of MA^0^ gas in both CsI and CBI occurs in the first 1 h, as shown in Figure S3 (Supporting Information). The absorbed MA^0^ molecules change the color of CBI powders from dark red to yellow and transform the powders from a solid state to a paste‐like state (Figure S4, Supporting Information). We also observed a slight decrease in the mass of the CBI‐MA^0^ powder sample after exposure to MA^0^ gas for 2 h. This reduction can be attributed to the easy release of absorbed MA^0^ molecules by the CBI as the concentration of MA^0^ gas in the system gradually decreases over time (more details are shown in the experimental section). As shown in Figure 4e, MA^0^ molecules can destroy the crystal structures of CBI and BiI_3_ powders, and both end up with similar final XRD patterns that correspond to the byproduct MAI (Figure S8, Supporting Information). Meanwhile, the XRD patterns of the CsI powder remain unchanged before and after the MA^0^ gas treatment, as shown in Figure S9 (Supporting Information). Correspondingly, there are no features in FTIR of CsI before and after exposure to MA^0^ gas (Figure S10, Supporting Information), confirming that MA^0^ molecules do not interact with CsI. In contrast, new features emerged in MA^0^‐treated CBI and BiI_3_ powders belonging to MAI (906, 990, 1246, 1402, 1484 cm^−1^), as shown in Figure 4f.^[^ ^32^ ^]^

Overall, Bi^3+^ is crucial for the absorption of MA^0^ in both Bi‐based perovskite systems. This is different from the organic–inorganic lead‐based halide perovskites, where the liquefaction of MAPbI_3_ can be attributed to two factors: the formation of hydrogen bonding between MA^0^ and MA^+^, and the bonding of MA^0^ to the Pb^2+^ atom via its lone electron pair.^[^ ^32^, ^33^ ^]^ While in Bi‐based inorganic perovskite systems, the underlying reason is supposed to be that MA^0^ molecules could coordinate with Bi^3+^ by occupying the empty Bi^3+^ 6p orbital (6s^2^6p^0^) through the lone electron pair of the nitrogen atom in MA^0^ (2s^2^), thus destroying the initial perovskite structure. To further verify this conclusion, we also employ other amine gases such as ethylamine (EA^0^) and butylamine (BA^0^) to treat CABB and CBI films. As shown in Figures S11 and S13 (Supporting Information), both amines can cause reversible optical bleaching in CABB and CBI films, similar to MA^0^. It is worth mentioning that the XRD peak intensities and grain orientation of EA^0^ and BA^0^‐treated CABB films were further enhanced than those of MA^0^‐treated films (Figure S12, Supporting Information). The reason is possibly related to the larger molecular size of BA^0^ and EA^0^ than MA^0^, which could destroy the initial perovskite structure more effectively and completely. In addition, this MA^0^‐induced optical bleaching is also observed in Cs_3_Sb_2_I_9_ (Figure S14, Supporting Information), which contains Sb^3+^ ions with electronic configurations akin to those of Bi^3+^, further reinforcing the conclusion and highlighting the generality of this approach.

**Figure 5:**
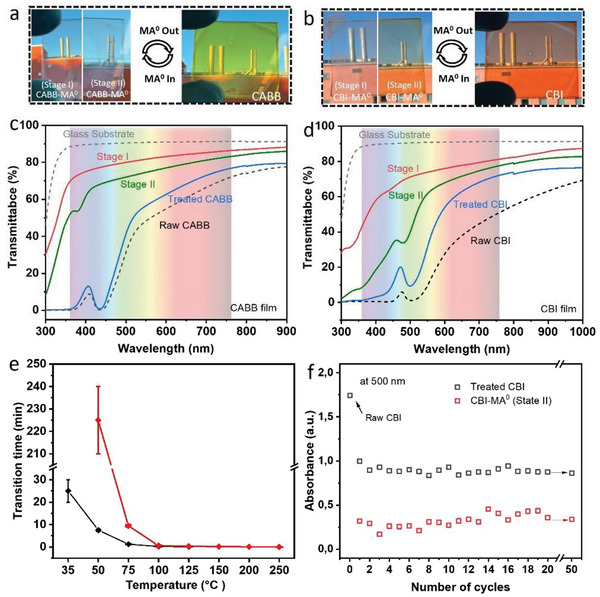
Photographs of smart windows based on a) CABB film and b) CBI film in their transparent and colored stages. Objects are visible through the windows in both stages. The transmittance spectra of the CABB c) and CBI d) films at different stages during MA^0^ treatment. The transmittance of the glass substrate is also given for reference. The colored background represents the visible light region from 360 to 760 nm. e) Variation of the transparent to color phase transition time of CBI and CABB film under different temperatures. f) The cycling stability of the absorbance at 500 nm in CBI film.

Encouraged by the remarkable and reversible optical feature changes between transparent and color stages of thin films, we evaluate their potential in smart window applications. **Figure** **5**a,b provide visual evidence of the distinct appearance of the transparent and colored (yellow or red‐brown) Bi‐based perovskite films (≈250–300 nm thickness), which have the potential to control the amount of visible light and heat that enter a room. The surface morphology of these films is characterized by SEM in Figure S15 (Supporting Information). We further evaluate the transmittance spectra of CABB and CBI films at different stages to assess their capacity for light manipulation. For CABB film under transparent stage, the average degree of visible (360–760 nm) transparency is ≈81.1% and 73.4% for Stage I and Stage II, respectively. These high average degrees of visible transparency can be reduced to 46.2% for the treated color CABB film (39.9% for raw CABB film) (Figure 5c). It should be noticed that the yellow CABB film has a negligible absorption above 600 nm due to its large bandgap of ≈2 eV. Thus, CABB film shows a better light‐adjusting ability in the 360–600 nm range, reducing the average transparency from 78.6% (in Stage I film) to 29.7% (in treated color film). Figure 5d shows the transmittance spectra of CBI film at different stages. The average degree of visible (360–760 nm) transparency for transparent stage I film is 71.3%, which can be significantly decreased to 36.6% for treated CBI film. The Stage II film with light yellow shows an average degree of visible transparency of 52.9%. We also noticed an obvious difference in visible transparency between the raw CBI film (19.9%) and treated CBI film (36.6%), consistent with the reduced absorption caused by morphology changes.

We also test the phase transition time from transparent state to colored state under different temperatures. As shown in Figure 5e, both CABB and CBI films show strong temperature‐dependent phase transition time. CBI demonstrates a notably higher transition rate than CABB, particularly at low temperatures below 75 °C. Interestingly, 75 °C could be considered a threshold temperature for both Bi‐based films, as temperatures above this threshold resulted in a significant reduction in transition time to just several minutes, or even seconds (Figure S16, Supporting Information). This could be explained by the weak bonding between MA^0^ and Bi^3+^ which can be broken at 75 °C, releasing absorbed MA^0^ molecules from the film. In the low‐temperature range below 50 °C, the MA^0^ molecules can remain in the CABB film for several days at room temperature, implying its potential use for data encryption applications (Figure S17, Supporting Information). In contrast, CBI demonstrated a short transition time of 20–30 min and 7–8 min for 35 and 50 °C, respectively. It is worth noting that this low phase transition temperature (35–50 °C) of CBI could potentially be achieved with sunlight as the heating source without requiring extra heating. Thus, the phase transition processes of CBI could be potentially leveraged where sunlight is used to heat the film and drive off MA^0^ gas with a transition to the colored perovskite phase, while the subsequent reintroduction of MA^0^ gas results in a phase transition back to the transparent phase.

Lastly, we investigate the cycling, working, and environmental stability of these Bi‐based films. Figure S18 (Supporting Information) shows the cycle‐dependent UV–vis absorption spectra of CABB film, where the transparent stage exhibits greater stability than the colored stage. Refining the focus to the characteristic peak position at ≈437 nm, the absorbance at colored stage gradually decreases (Figure S18b, Supporting Information). Additionally, several impurity diffraction peaks appeared in CABB film after 20 cycles of phase transitions (Figure S19, Supporting Information). For CBI film, the first time MA^0^ treatment reduces the absorbance of the raw film by nearly half due to the dramatic change in film morphology (Figure S20a, Supporting Information), consistent with the transmittance spectra in Figure 5d. After that, the absorbance of the CBI film is stable with almost no change after 50 cycles, indicating the excellent cycle stability of CBI film (Figure 5f; Figure S20, Supporting Information). Meanwhile, there are also no other impurity peaks in the XRD patterns after 50 cycles of phase transitions (Figure S20b, Supporting Information). Regarding the working stability, both CABB and CBI films can recover their original color after 40 h of MA^0^ gas exposure, as shown in Figure S21 (Supporting Information). The CABB film exhibits a reduction in absorbance and the emergence of an impurity peak in XRD after 20 h of exposure, whereas the CBI film demonstrates superior working stability with only slight changes in absorbance. Furthermore, both MA^0^‐treated CABB and CBI films display excellent environmental stability, evidenced by negligible XRD changes after a prolonged air exposure of 180 days (Figure S22, Supporting Information). When considering applications in smart windows, the CBI film shows more promise due to its better capability to modulate visible light, lower phase transition temperature, short phase transition time, and better overall stability in cycling and operation.

## 3 Conclusion

In summary, we demonstrate a reversible optical bleaching phenomenon in lead‐free Bi‐based halide perovskite thin films through the absorption and release of MA^0^ gas molecules, resulting in a significant color change from yellow and red to transparent for CABB and CBI, respectively. XRD, UV–vis, Raman, and SEM results indicate that the MA^0^ molecules induce a full collapse of the initial perovskite structure to a liquefied transparent intermediate phase by the interaction between the MA^0^ molecules and Bi^3+^, leading to optical bleaching. This intermediate phase can recrystallize back to the perovskite phase with improved orientation and morphology after removing MA^0^ molecules from the film. The CBI film shows a wider chromatic variation between the transparent stage (71.3% average visible transparency) and dark‐red colored stage (36.6% average visible transparency), lower phase transition temperatures (35–50 °C), shorter phase transition time (below 30 min), superior phase transition cycle stability (more than 50 cycles) and better working stability (more than 40 h) than CABB film, attractive for smart windows and other on‐demand applications. In contrast, the MA^0^‐treated CABB film exhibits significantly improved grain orientation, enhanced crystallinity, and uniform morphology, highly desirable for some optoelectronic applications such as solar cells, photo/X‐ray detectors, etc. In addition, other amine gases including EA^0^ and BA° can also introduce optical bleaching in CABB, CBI, and Cs_3_Sb_2_I_9_ films, suggesting the universality of the interaction between amine gases and inorganic perovskites. Our finding opens up the great potential to exploit gas‐solid reactions in lead‐free perovskites.

## 4 Experimental Section

### Materials and Films Fabrication

All the chemicals used were purchased from Sigma–Aldrich without any further purification. Microscope slides as glass substrates were purchased from Menzel Gläser. To prepare CABB thin films, a 0.5 m precursor solution was made by dissolving CsBr (212.8 mg), AgBr (93.88 mg), and BiBr_3_ (224.35 mg) powder in 1 mL of dimethyl sulfoxide (DMSO), which was then heated at 100 °C and stirred for 12 h until a clear yellow solution was obtained. Afterward, 45 µL of the precursor solution was spin‐coated onto a glass substrate at 3000 rpm for 60 s with an N_2_ flow into the spin coater chamber in an ambient environment. The substrates were annealed at 280 °C in air for 5 min to produce high‐quality CABB films. For the preparation of CBI thin films, a 0.25 m CBI precursor solution was made by dissolving CsI (97.43 mg) and BiI_3_ (147.4 mg) powder in 0.4 mL DMF and 0.1 mL DMSO solvent. The solution was heated to 60 °C and stirred for above 12 h, and then filtered through a 0.45 µm PTFE filter to obtain a clear solution. Subsequently, 45 µL of the precursor solution was spin‐coated onto glass at 3000 rpm for 60 s in an ambient environment with N_2_ flown into the spin coater chamber. Finally, the substrates were annealed at 125 °C for 30 min to produce high‐quality CBI films. For the preparation of Cs_3_Sb_2_I_9_ thin films, 0.75 m CsI and 0.5 m SbI_3_ powder were dissolved in a mixed solvent (DMF:HCl, 1:0.03 volume ratio). Afterward, 45 µL of the precursor solution was spin‐coated onto a glass substrate at 3000 rpm for 60 s. The substrates were annealed at 230 °C for 10 min to produce high‐quality films.

### Amine Gases Generation and Treatment

MA^0^ gas atmosphere was easily obtained by adding 5–10 mL methylamine solution (33 wt.% in absolute ethanol) to a 200 mL glass beaker, which was then covered with aluminum foil before and after MA^0^ treatment to maintain the MA^0^ gas concentration. All processes were done at room temperature and under normal pressure in a fume hood. The film was placed face down into the beaker vertically, ≈2–3 cm from the liquid surface, and stayed for 2–3 s until the color bleaches, after which the film was removed vertically from the beaker. Normally, to accelerate MA^0^ gas removal, CABB and CBI films were annealed at 250 and 125 °C for 2 min, respectively. The EA^0^ and BA^0^ gas atmospheres were generated in a similar method to MA^0^, using ethylamine (2.0 m in methanol) and butylamine (99.5%) solutions, respectively. Other processes were the same as MA^0^ treatment.

For the experiment investigating MA^0^ absorption behavior with halide salts and perovskite powders, the MA^0^ gas was generated by mixing MACl and KOH powders in 5 mL glass bottles, with CaCl_2_ powder placed on top for drying. The precursor powders were also stored separately in 5 ml bottles. Then, all the above bottles were transferred to one large 100 mL glass bottle to allow precursor powders to absorb MA^0^ gas molecules continuously. The mass differences of powders with different exposure times were tracked to investigate the absorption behavior. CsBr, AgBr, BiBr_3_, CsI, and BiI_3_ powders were directly obtained by grinding the corresponding commercial chemicals from Sigma–Aldrich. CABB powder was obtained by grinding CABB single crystals, which were prepared using a hydrothermal method described in the previous report.^[^ ^34^ ^]^ For CBI powder, it was collected from the hot‐casting CBI film by dropping precursors onto a glass substrate and annealing at 125 °C for 2 h.

### Materials and Films Characterization

The XRD patterns of the powders and thin films were carried out using a Siemens D5000 θ–2θ goniometer with Cu Kα1 irradiation (λ = 1.5406 Å). The UV–vis absorption and transmittance spectra were measured via a Cary 5000 spectrophotometer. The FTIR spectra were recorded on powder samples on a Bruker (Vertex 70v) spectrometer. The surface and cross‐sectional SEM images were taken using ZeissLEO1530/1550 microscopes. Raman spectra measurements were performed in backscattering geometry by using a confocal Horiba Jobin–Yvon HR800 system. The excitation light was produced by a solid‐state 660 nm laser and the incident power density was set as < 1 mW µm^−2^ to avoid heating effects.

## Conflict of Interest

The authors declare no conflict of interest.

## Supporting information

Supporting Information

## Data Availability

The data that support the findings of this study are available from the corresponding author upon reasonable request.
