# Supplementary material for: Amine Gas‐Induced Reversible Optical Bleaching of Bismuth‐Based Lead‐Free Perovskite Thin Films
Source: Adv Sci (Weinh). 2023 Dec 3;11(4):2306391. doi: 10.1002/advs.202306391 (PMC10811464; doi:10.1002/advs.202306391)
Supplement: Supplementary file 1 — Supporting Information [file ADVS-11-2306391-s001.pdf]

ADVANCED SCIENCE
Open Access

Supporting Information

for *Adv. Sci.*, DOI 10.1002/advs.202306391

Amine Gas-Induced Reversible Optical Bleaching of Bismuth-Based Lead-Free Perovskite Thin Films

Fuxiang Ji, Bin Zhang, Weimin M Chen, Irina A Buyanova, Feng Wang and Gerrit Boschloo**

Amine gases-induced reversible optical bleaching of bismuth-based lead-free perovskite thin films

Fuxiang Ji,[a*] Bin Zhang,[b] Weimin M Chen,[b] Irina A Buyanova,[b] Feng Wang,[b] Gerrit Boschloo[a*]

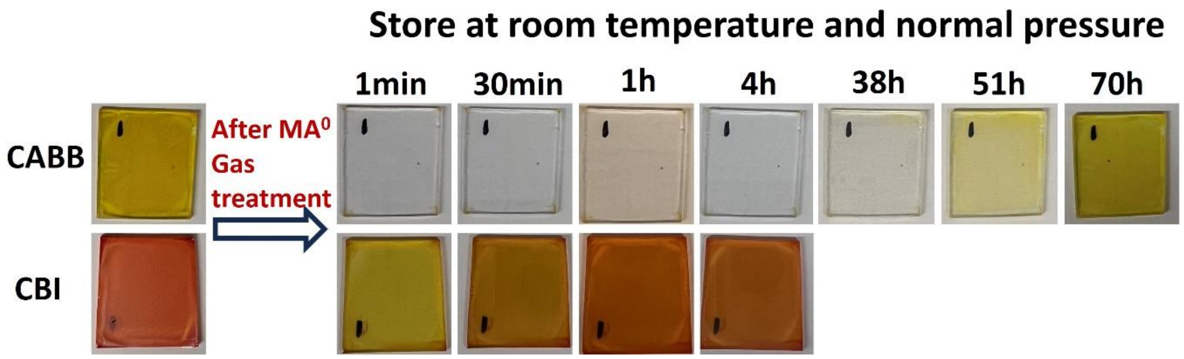

Figure S1. Optical images of CABB and CBI films stored at room temperature and normal pressure after treatment with MA^0 gas.

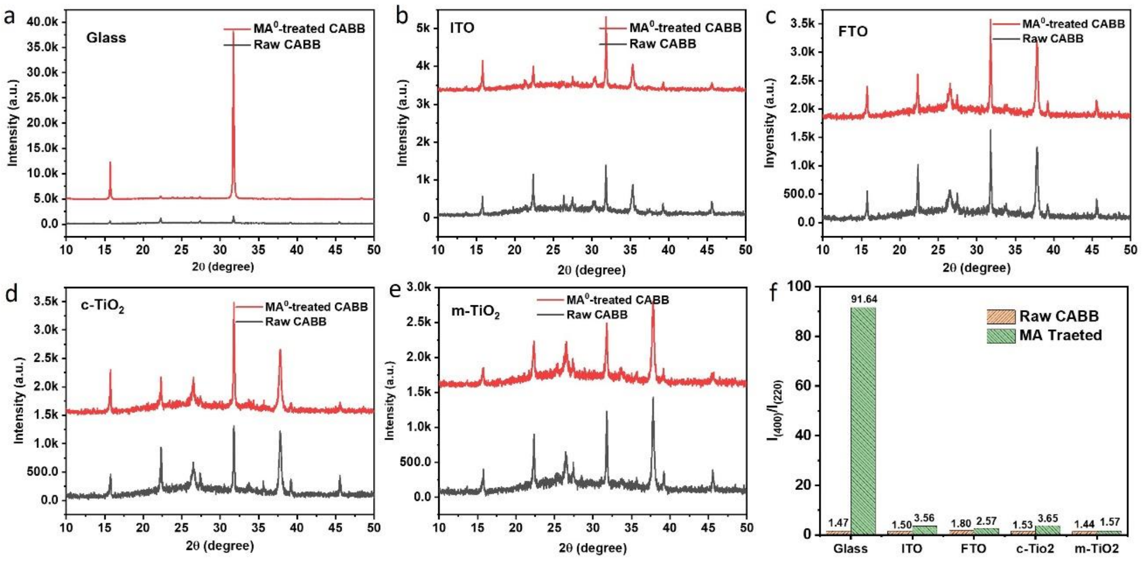

Figure S2. XRD patterns of CABB film on different substrates before and after MA^0 treatment, including glass (a), ITO (b), FTO (c), compact TiO_2 (d), and mesoporous TiO_2 (e). (f) The diffraction intensity ratio between the (400) and (220) peaks in raw and MA^0-treated CABB films on different substrates.

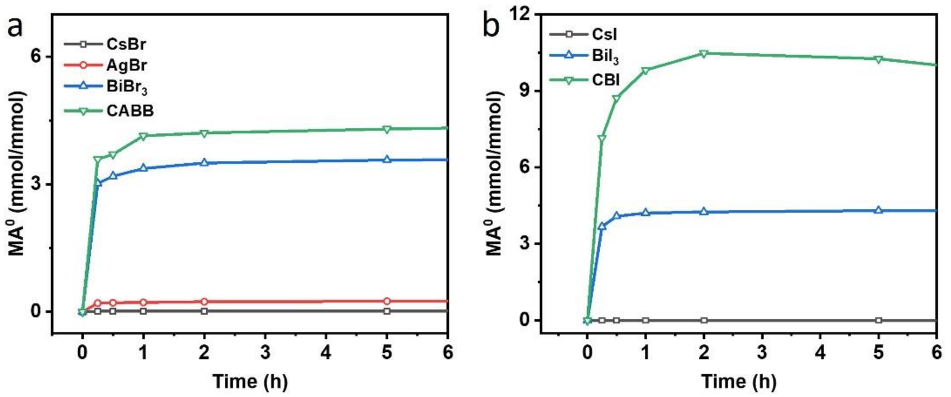

Figure S3. MA^0 gas absorption behavior of different halide salts and perovskite powder in CABB (a) and CBI (b) at the first 6 hours of MA^0 exposure.

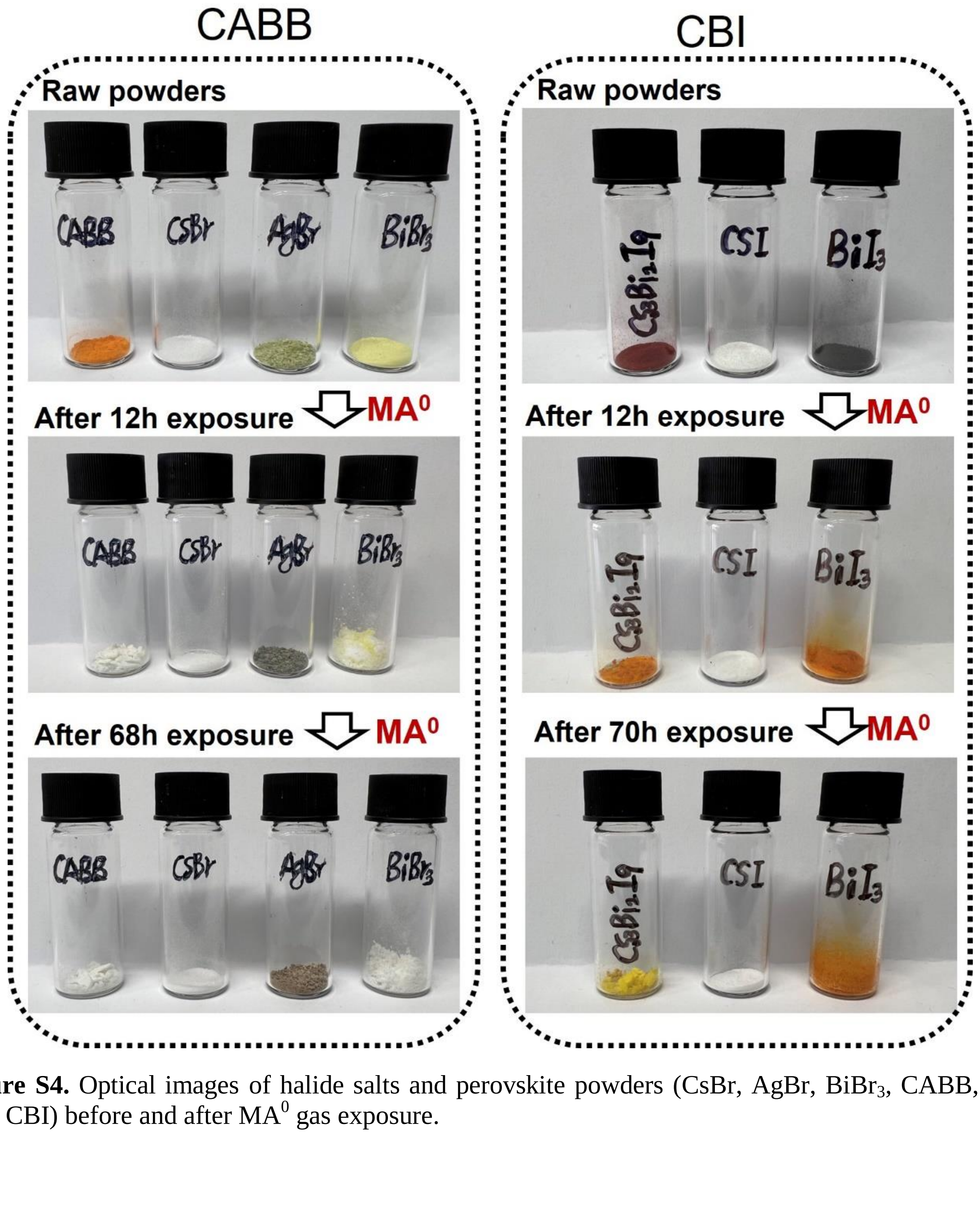

Figure S4. Optical images of halide salts and perovskite powders (CsBr, AgBr, $BiBr_3$, CABB, CsI, BiI_3, CBI) before and after MA^0 gas exposure.

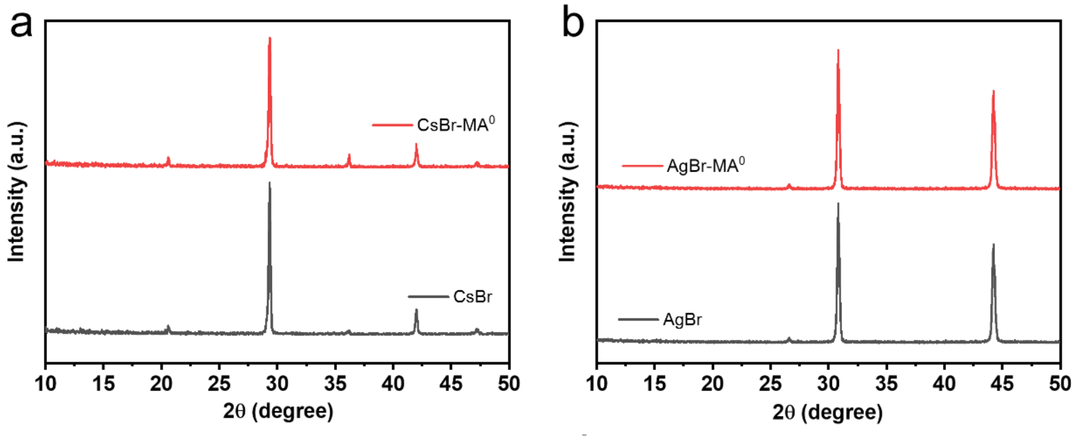

Figure S5. XRD patterns of CsBr (a) and AgBr (b) powders before and after absorbing MA^0 gas molecules.

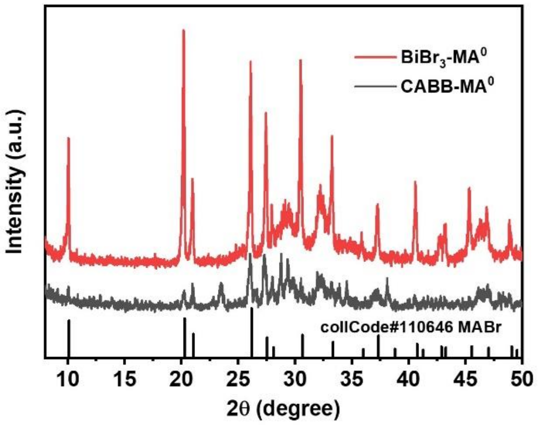

Figure S6. XRD patterns of CABB and $BiBr_3$ powders after absorbing MA^0 gas molecules.

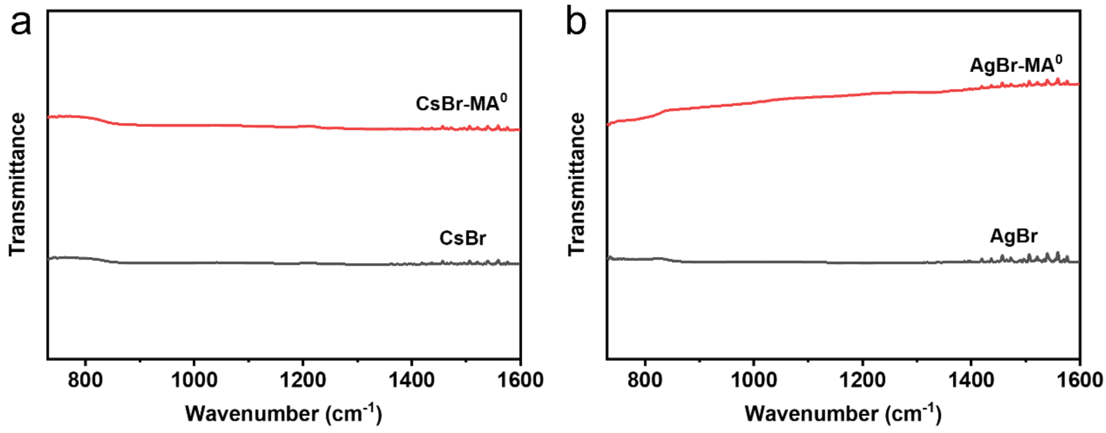

Figure S7. FTIR spectra of CsBr (a) and AgBr (b) powders before and after absorbing MA^0 gas molecules.

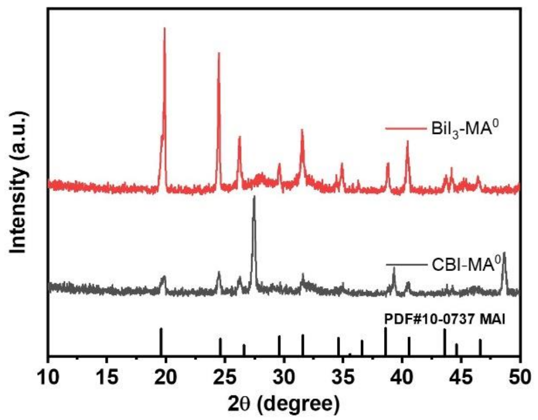

Figure S8. XRD patterns of CBI and BiI_3 powders after absorbing MA^0 gas molecules.

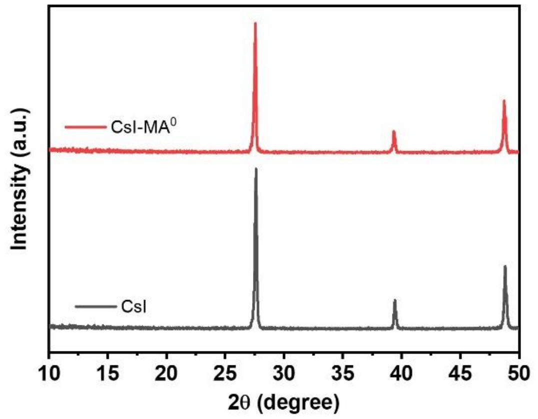

Figure S9. XRD patterns of CsI before and after absorbing MA^0 gas molecules.

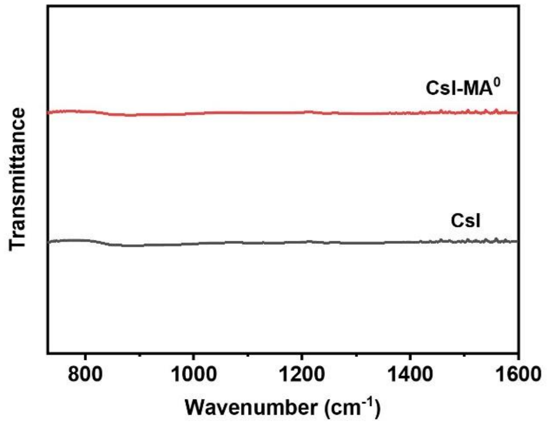

Figure S10. FTIR spectra of CsI powders before and after absorbing MA^0 gas molecules.

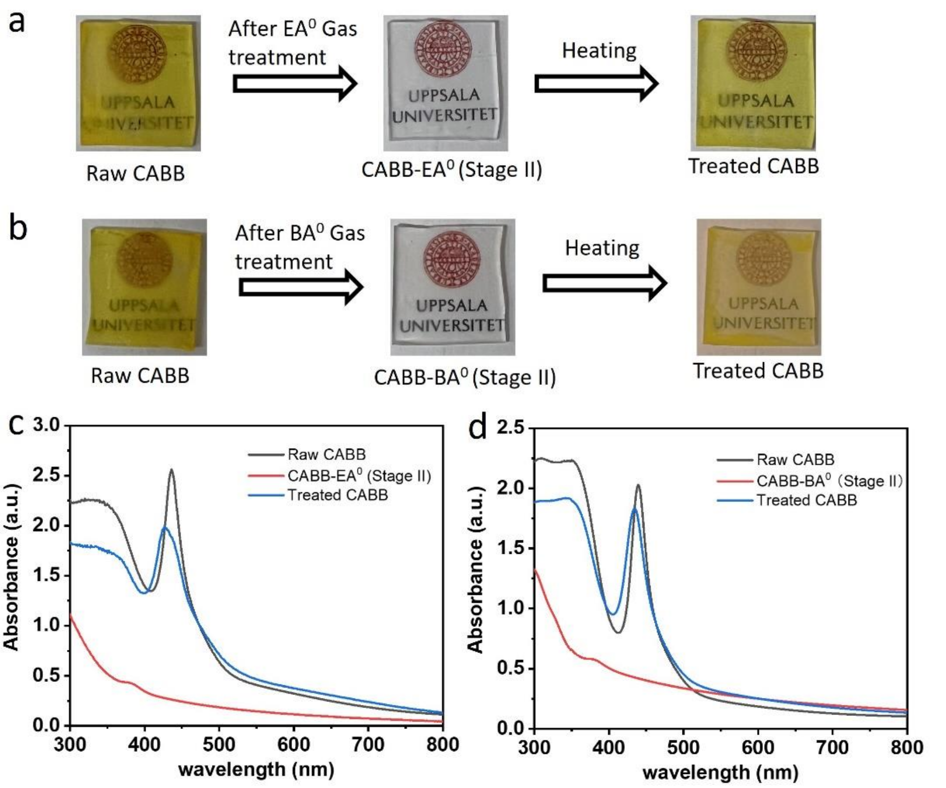

Figure S11. Optical images of optical bleaching in CABB film during EA^0 (a) and BA^0 (b) gas treatment. UV-vis absorption spectra of CABB film at different states during EA^0 (c) and BA^0 (d) gas treatment.

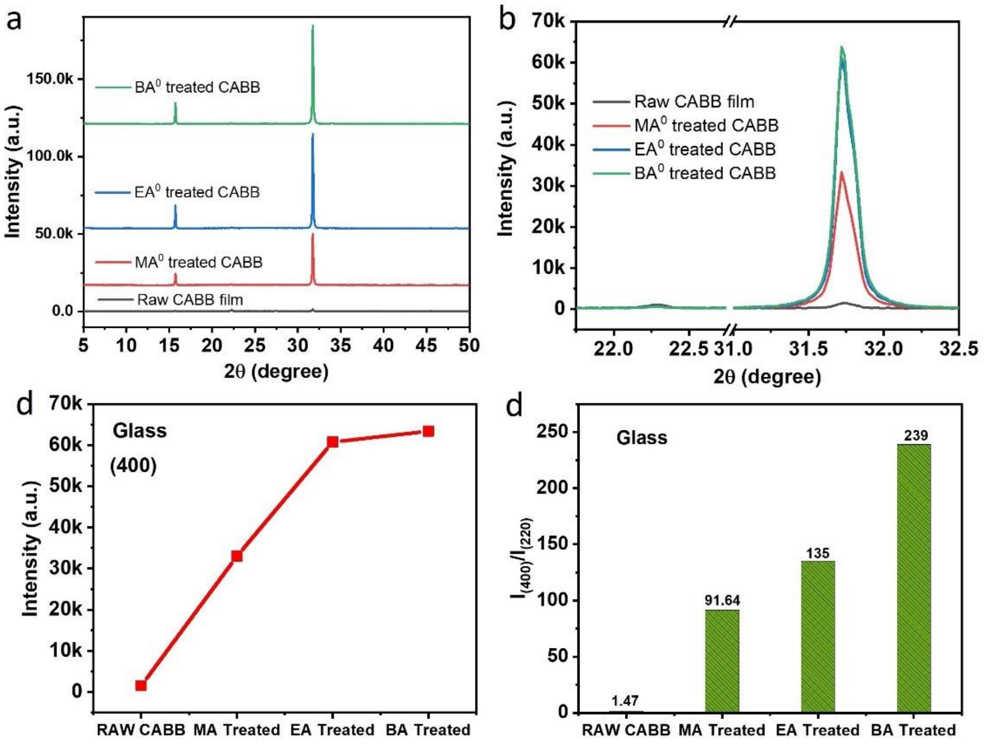

Figure S12. (a) XRD patterns of CABB film after different amines treatment. (b) The enlarged view of the (220) and (400) diffraction peaks in the XRD patterns. (c) The intensity of (400) diffraction peak in different amine gases treated CABB films. (d) The diffraction intensity ratio between the (400) and (220) peaks in different amine gases treated CABB films.

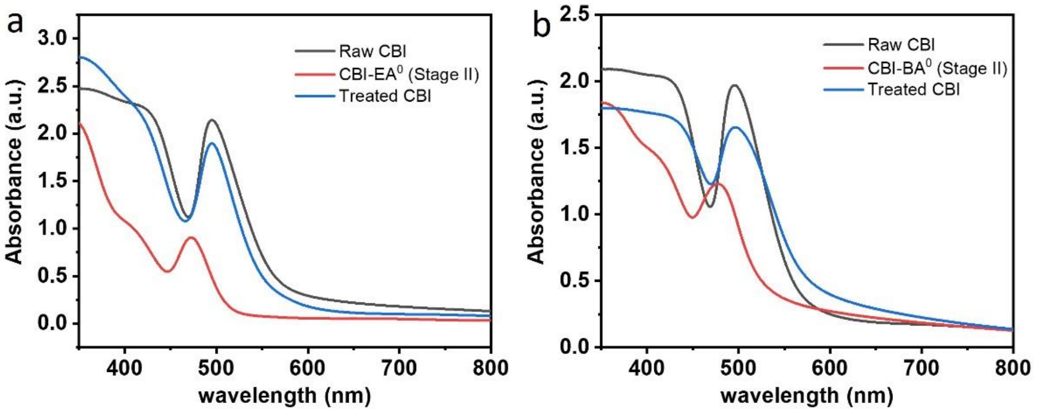

Figure S13. UV-vis absorption spectra of CBI film at different stages during EA^0 (a) and BA^0 (b) gas treatment.

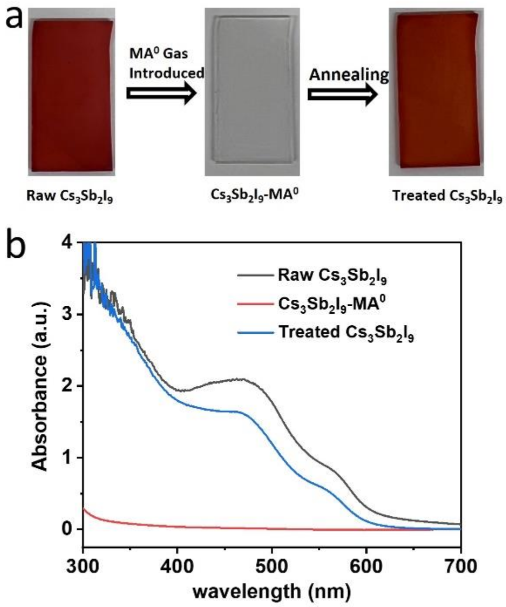

Figure S14. (a) Optical images of optical bleaching in $Cs_3Sb_2I_9$ film during MA^0 gas treatment. (b) UV-vis absorption spectra of $Cs_3Sb_2I_9$ film at different states during MA^0 gas treatment.

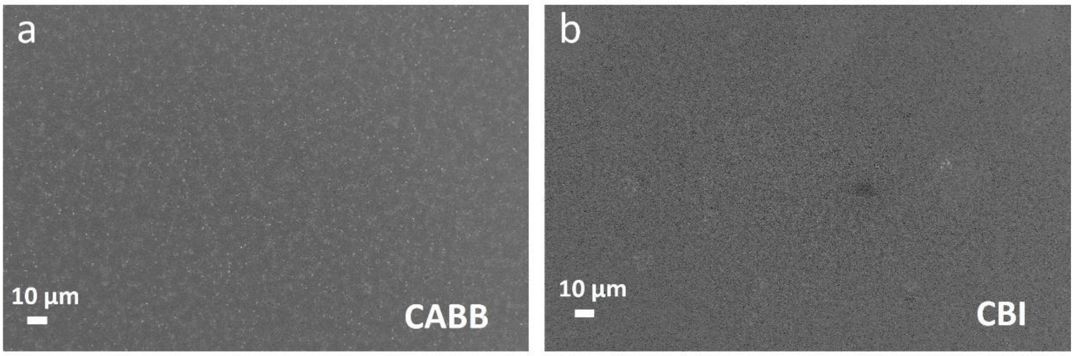

Figure S15. Large-scale SEM images of MA^0-treated large-area CABB (a) and CBI (b) film.

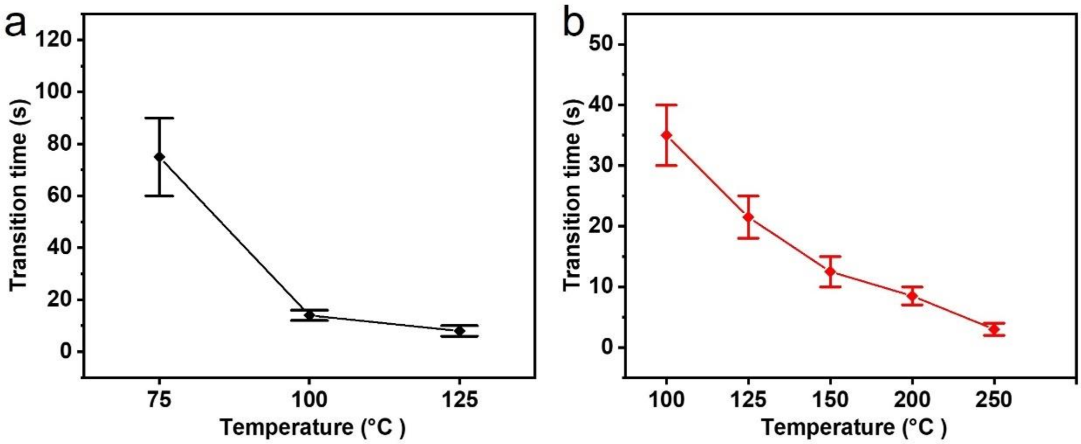

Figure S16. Variation of the transparent to color phase transition time of CBI and CABB film under different temperatures.

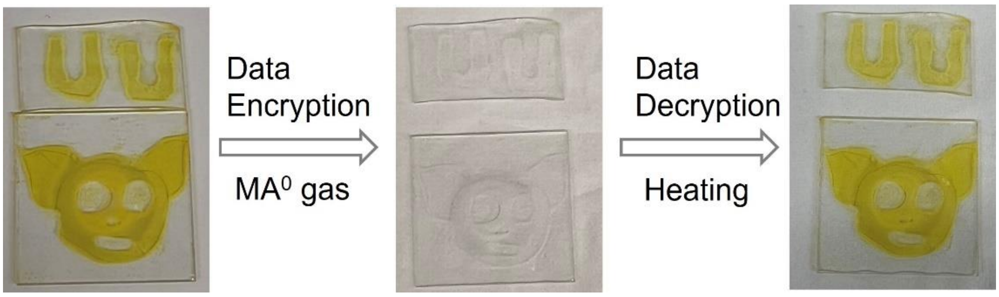

Figure S17. Optical images of data encryption and decryption process based on CABB film and MA^0 gas.

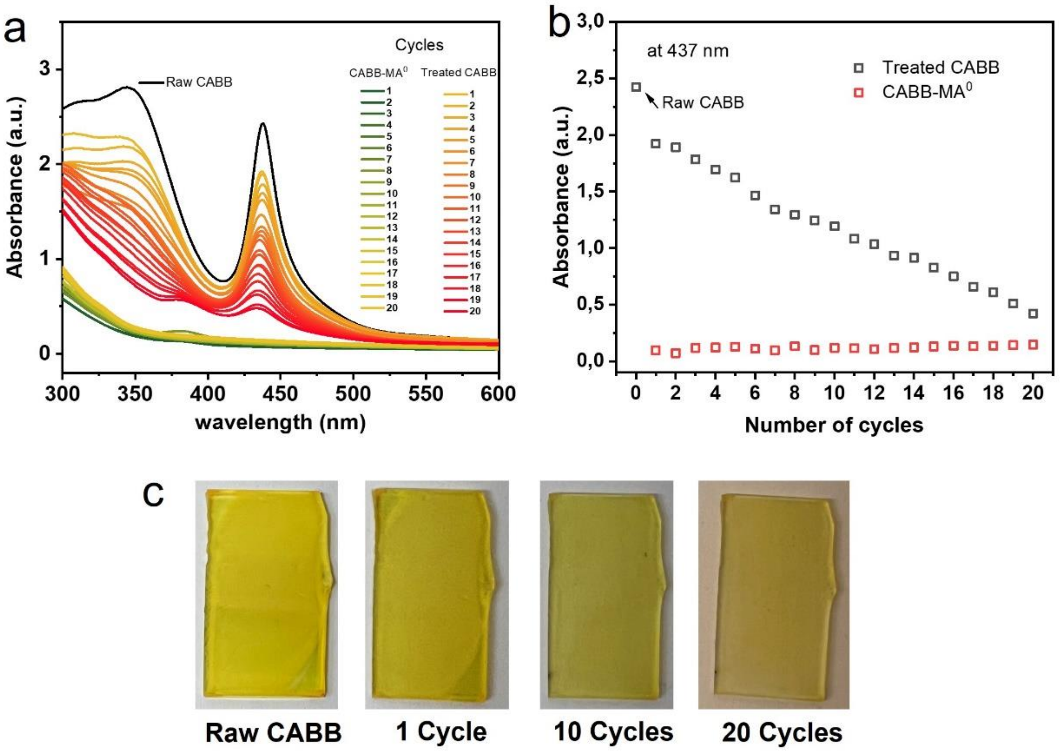

Figure S18. (a) UV-vis absorption spectra of CABB film at transparent (State II) and colored states for repeating 20 cycles. (b)The cycling stability of the absorbance at 437 nm in CABB film. (c) Optical images of CABB film after 1-20 cycles of phase transitions.

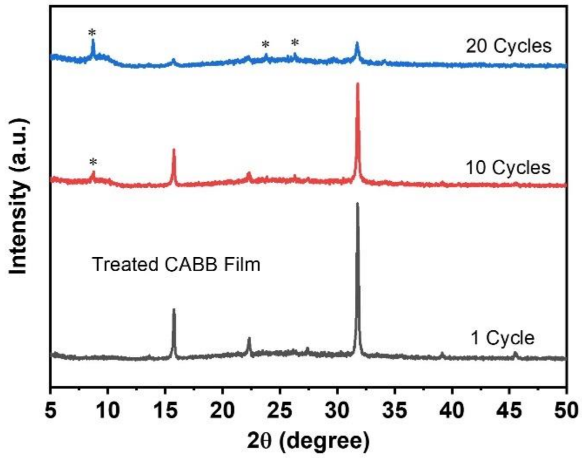

Figure S19. XRD patterns of CABB film after 1, 10 and 20 cycles of phase transitions. Asterisks represent impurity peaks.

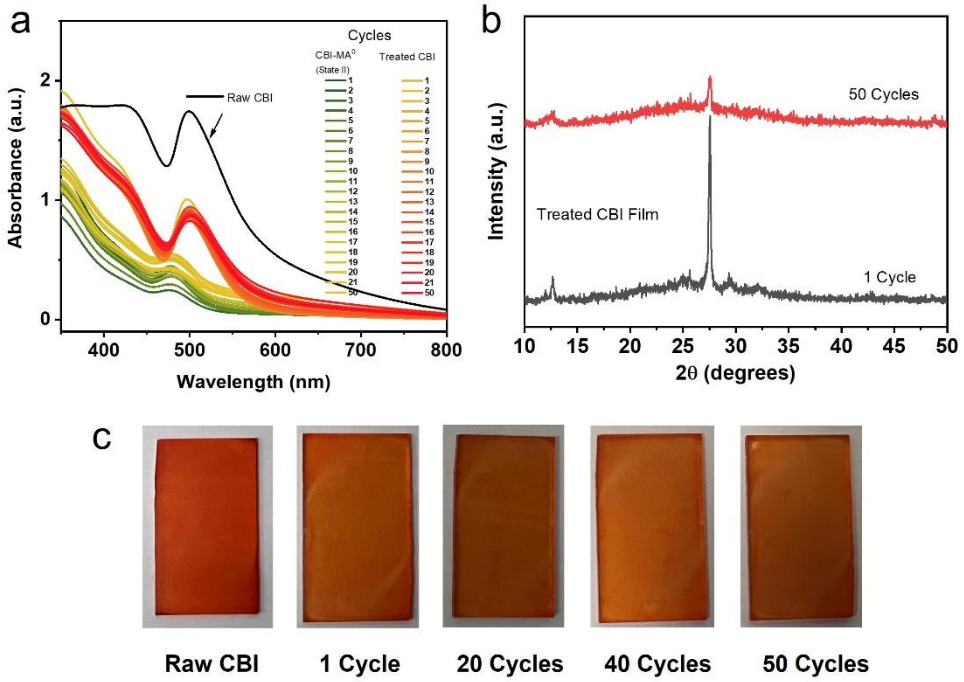

Figure S20. (a) UV-vis absorption spectra of CBI film at transparent (State II) and colored states for repeating 50 cycles. (b) XRD patterns of CBI film after 1 and 50 cycles of phase transitions. (c) Optical images of CBI film after 1-50 cycles of phase transitions.

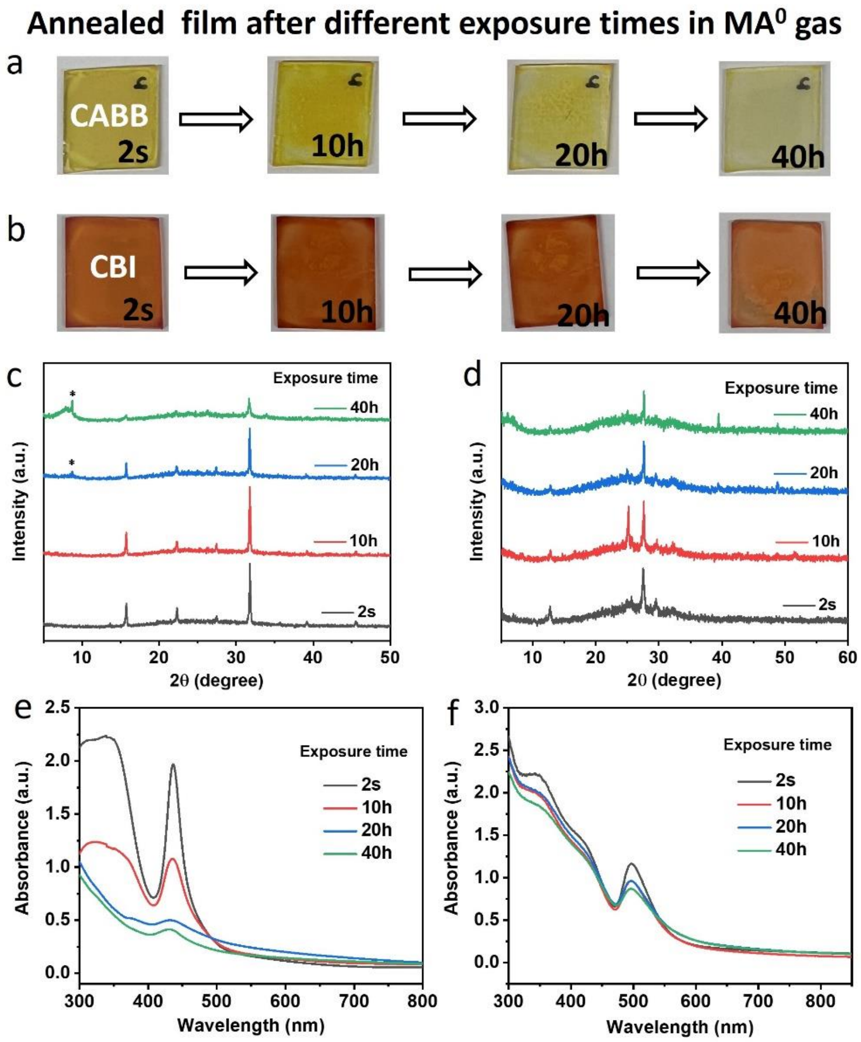

Figure S21. Optical images of annealed CABB (a) and CBI (b) films after 2 seconds to 40 hours of MA^0 gas exposure. XRD patterns of annealed CABB (c) and CBI (d) film after 2s-40h of MA^0 gas exposure. UV-vis absorption spectra of annealed CABB (e) and CBI (f) film after 2s-40h of MA^0 gas exposure.

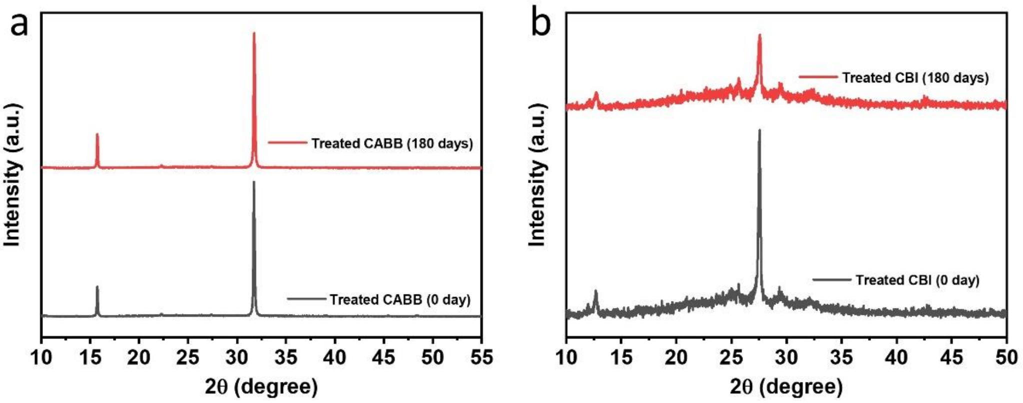

Figure S22. XRD patterns of CABB (a) and CBI (b) film before and after 180 days surrounding environment storage.
